# Genome Wide Re-Annotation of *Caldicellulosiruptor saccharolyticus* with New Insights into Genes Involved in Biomass Degradation and Hydrogen Production

**DOI:** 10.1371/journal.pone.0133183

**Published:** 2015-07-21

**Authors:** Nupoor Chowdhary, Ashok Selvaraj, Lakshmi KrishnaKumaar, Gopal Ramesh Kumar

**Affiliations:** AU-KBC Research Centre, Anna University, MIT Campus, Chrompet, Chennai, 600044, India; Missouri University of Science and Technology, UNITED STATES

## Abstract

*Caldicellulosiruptor saccharolyticus* has proven itself to be an excellent candidate for biological hydrogen (H_2_) production, but still it has major drawbacks like sensitivity to high osmotic pressure and low volumetric H_2_ productivity, which should be considered before it can be used industrially. A whole genome re-annotation work has been carried out as an attempt to update the incomplete genome information that causes gap in the knowledge especially in the area of metabolic engineering, to improve the H_2_ producing capabilities of *C*. *saccharolyticus*. Whole genome re-annotation was performed through manual means for 2,682 Coding Sequences (CDSs). Bioinformatics tools based on sequence similarity, motif search, phylogenetic analysis and fold recognition were employed for re-annotation. Our methodology could successfully add functions for 409 hypothetical proteins (HPs), 46 proteins previously annotated as putative and assigned more accurate functions for the known protein sequences. Homology based gene annotation has been used as a standard method for assigning function to novel proteins, but over the past few years many non-homology based methods such as genomic context approaches for protein function prediction have been developed. Using non-homology based functional prediction methods, we were able to assign cellular processes or physical complexes for 249 hypothetical sequences. Our re-annotation pipeline highlights the addition of 231 new CDSs generated from MicroScope Platform, to the original genome with functional prediction for 49 of them. The re-annotation of HPs and new CDSs is stored in the relational database that is available on the MicroScope web-based platform. In parallel, a comparative genome analyses were performed among the members of genus *Caldicellulosiruptor* to understand the function and evolutionary processes. Further, with results from integrated re-annotation studies (homology and genomic context approach), we strongly suggest that Csac_0437 and Csac_0424 encode for glycoside hydrolases (GH) and are proposed to be involved in the decomposition of recalcitrant plant polysaccharides. Similarly, HPs: Csac_0732, Csac_1862, Csac_1294 and Csac_0668 are suggested to play a significant role in biohydrogen production. Function prediction of these HPs by using our integrated approach will considerably enhance the interpretation of large-scale experiments targeting this industrially important organism.

## Introduction

Currently, fossil fuels such as coal, oil and natural gas are the major sources of global energy requirements (comprising of about 95%). Industries mine for these fossil fuel resources to produce electricity or refine them to be utilized as fuel. The fossil fuel reserves are finite and their extensive utilization is held responsible for changes in worldwide atmosphere, bringing about emanation of pollutants [1]. Renewable energy resources are suggested as an alternative which are capable of overcoming these problems. H_2_ is considered to be the most promising future fuel as it is non-polluting in nature and burns to form water, which can be further reused [2,3]. H_2_ production from biomass is a characteristic of prokaryotes and is considered to be an essential wellspring of renewable energy [4]. Conventional or first generation biofuels are derived from sources like sugar, starch or vegetable oils that are edible and contend with the human utilization, inevitably increasing the market value. As a result, greater accentuation is to be put on second-generation biofuels that are produced from wide variety of feedstocks particularly from non edible lignocellulosic biomass and domestic as well as industrial wastes [5].

Biohydrogen is considered as the cleanest fuel with no emission of greenhouse gases on combustion. Different biological processes such as dark fermentation and photo fermentation can be used to produce H_2_ from lignocellulosic biomass and waste materials [6,7,8]. However a combination of these two processes, i.e. dark fermentation and photo fermentation is an interesting approach for the maximum conversion of energy from carbohydrate rich substrates [9]. Maximum of 12 moles of H_2_ can be obtained in the complete oxidation of per mole of glucose. However, theoretically only 4 moles of H_2_ can be obtained per mole of glucose through dark fermentation process and this yield is obtained when the partial pressure of H_2_ (pH2) is kept adequately low [9,10]. Issues like low H_2_ yield from different organisms and the requirement for low pH2, should be managed for large scale biohydrogen production [9,11].

*Caldicellulosiruptor saccharolyticus* gained interest due to its property to produce high H_2_ yield (maximum 3.3 to 3.6 mol H_2_ per mol glucose), its insensitivity to high pH2 and its ability to hydrolyse variety of polymeric carbohydrates. *C*. *saccharolyticus* is extremely thermophilic, strictly anaerobic asporogenous bacterium that was isolated from the Rotorua-Taupa, thermal area in New Zealand [12]. It can grow at a temperature range of 45–80°C (Topt = 70°C) and pH range of 5.5–8.0 (pHopt = 7.0). Several cellulolytic *Caldicellulosiruptor* species have been isolated from various thermal springs and fully sequenced genomes of eight of these *Caldicellulosiruptor* species are available now [12].

The massive generation of genomic data from the next generation sequencing (NGS) platforms has also increased the errors in genome annotation, since they are annotated automatically and deposited in the public domain databases [13]. Currently, only few genes in prokaryotes are wet lab characterized for e.g. in *C*. *saccharolyticus* β-glucosidase (BglA) [14], β-xylosidase [15], β-1,4-xylanase [16], type I pullulanase [17] and beta-mannanase/endoglucanase A (manA) [18] have been cloned into *Escherichia coli* and characterized. All other genes information is extracted by using conventional tools and submitted into the databases. Therefore, re-annotation is an important step where each gene is reviewed manually with the aim of improving its product description. The complete genome of *C*. *saccharolyticus* was first sequenced and annotated in 2008 [4]. The genome consists of one 2,970,275-bp circular chromosome, which has a G+C content of 35.3% and 2,679 (2,682 in NCBI) protein encoding genes. However, only 1,854 CDSs have been functionally annotated while the remaining 828 sequences were classified into either hypothetical or putative category. Hypothetical protein (HP) is a protein that is predicted to be expressed from an open reading frame, but for which there is no experimental evidence of translation available. Similarly, putative proteins share similarity to an extent with characterized proteins in their conserved amino acid residues region and no other significant region will be similar to annotated proteins [19]. For the re-annotation purpose, we have combined the sequential information of proteins with functional domain identification and evolutionary studies using genomic data from closely related species [20,21]. Furthermore, Receiver Operating Characteristic (ROC) analysis [22] was additionally carried out for evaluating the performance of used bioinformatics tools.

This study describes the methodology for manual re-annotation with updation of all the CDSs of *C*. *saccharolyticus* and we combine homology based function prediction as well as genomic context approaches to reduce the number of HPs in *C*. *saccharolyticus*. Homology based function prediction of an unknown protein requires information transfer from experimentally characterized proteins, while genomic context based approach predicts functional associations based on physical interactions, gene neighborhood or significant co-occurrence of genes across different species [23,24,25]. With the aim of getting further insights into *C*. *saccharolyticus* new annotation and the availability of 7 complete genome sequences from the members of the same genus, prompted us to conduct a comparative study among them. This new genome annotation and comparative analyses will further help in the study of metabolic pathway networks of the organism which in turn will be useful in increasing the H_2_ production by metabolic engineering [20].

## Materials and Methods

### Sequence Retrieval

Genomic sequence of *Caldicellulosiruptor saccharolyticus* DSM 8903 in FASTA format was downloaded from NCBI-Microbial Genome Database (http://www.ncbi.nlm.nih.gov/nuccore/146295085?report=fasta) and was submitted to the Microbial genome annotation and analysis platform (MicroScope) for gene prediction [26].

### Identification of new CDSs in the *Caldicellulosiruptor saccharolyticus* genome

MicroScope was developed as a platform to support microbial genome re-annotation and comparative analysis [26]. MicroScope uses AMIGene (Annotation of Microbial Genes) [27] application to identify the most likely CDSs in the complete bacterial genome. AMIGene is based on Markov models. The first and the most essential step in AMIGene is the construction of Markov models that fit the input genomic data. Given a complete genome sequence, AMIGene first looks for maximal CDSs with maximal segment in frame between start and stop codon and then retains putative CDSs with base pairs greater than 60. During the re-annotation process, the newly predicted CDSs were assigned with a locus tag of “CALS8” followed by a four-digit number both separated by an underscore. The new CDSs were classified into two categories by the AMIGene tool: i) NEW, ii) UNIQUE_AMIGA. The “NEW” has high probability of encoding protein, while the “UNIQUE_AMIGA” CDSs are often like product with protein of unknown function or have a low probability of coding for functional protein. Further the protein sequences obtained from MicroScope were divided into five categories: i) Known, ii) Hypothetical, iii) Putative, iv) New CDSs (including NEW+UNIQUE_AMIGA), v) Pseudogenes or gene remnants.

### Re-annotation of the complete genome

Each of the predicted protein sequences were sequentially analyzed by searches like PSI-BLAST [28], BLASTO [29], Pfam 27.0 [30], InterProScan 5 [31] and ANNIE [32] (S1 and S2 Tables). We have used Position-Specific Iterative PSI-BLAST instead of BLASTp since it is more capable of detecting distant sequence similarities than a single query alone in the latter. The PSI-BLAST search was performed against the non-redundant protein sequences up to 3 iterations with maximum number of aligned sequences to be displayed for each HP selected as 100. The results obtained from PSI-BLAST with high sequence identity (>40%) and threshold e-value (<1*10^−52^) were chosen as best hits. BLASTO is an integrated platform that correlates orthology information into functional inference and evolutionary studies of individual sequences. It computes the significant score of each orthologous group based on their BLAST hits, using the number of taxa as optional weight. Protein families-based searches were carried out using Pfam that contains two components Pfam-A and Pfam-B. Pfam-A entries are high quality, manually curated families, whereas Pfam-B entries are of lower quality that contains a large number of small families derived from clusters produced by an algorithm called ADDA (Automatic Domain Decomposition Algorithm). The protein sequences were searched against Pfam-A families with an e-value of 1e-02. ANNIE and InterProScan are integrated resources that combine various useful algorithms for sequence-based analysis. Five levels of confidence (1/5 to 5/5) were assigned to each predicted CDS, depending on the number of tools that agreed on the function of the CDS. For integrated searches the source tool used for function prediction was also taken into consideration to avoid intrinsic overlaps during confidence level assignment.

Functional categorization of protein sequences were carried out based on COG functional classifications [33] with MicroScope. The prediction of functions of individual proteins or protein sets, is done by fitting proteins into the COG using the COGNITOR program. COGNITOR program assign proteins to COGs on the basis of multiple genome-specific best hits (BeTs) and also splits multi-domain protein into individual domain if they show affinity with different COGs. The cut-off for assigning proteins to COGs is set at three BeTs. A comparative analysis was performed based on COG classifications for the genomic distribution of proteins before and after re-annotation.

The list of minimal gene set was also generated for *C*. *saccharolyticus* using MicroScope platform. The list of essential genes was taken from Gil et al., 2004 [34]. The minimal gene sets were identified by performing homology searches between each gene of *C*. *saccharolyticus* and the set of 206 essential genes from 7 species (*Escherichia coli* K12, *Bacillus subtilis* 168, *Candidatus Blochmania floridanus*, *Buchnera aphidicola* APS, *Buchnera aphidicola* Bp, *Buchnera aphidicola* Sg and *Mycoplasma genitalium* G37). The candidate gene to be considered as essential gene has to fulfill any one of the following conditions, either it should share a Bidirectional best hits (BBH) relationship with a minLrap (>0.5) or it should belong to the same synteny group. BBH method is used in identifying the pairs of genes in two different genomes that are more similar to each other than either is to any other gene in the other genome. minLrap is the ratios of alignment lengths computed for comparison of the pair of genes using the BLAST software: minLrap = Lmatch/min (Lprot1, Lprot2), where Lmatch = length of the match, Lprot1 = length of protein 1, Lprot2 = length of protein 2.

If minLrap = 1 and maxLrap = 1 = > the 2 proteins both align on their whole length.

If minLrap = 1 and maxLrap<1 = > one of the proteins is longer than the other, or the alignment is partial.

If minLrap<1 and maxLrap<1 = > the sequences are poorly aligned.

Sub-cellular localization prediction tool PSORTb [35] was used for HPs localization information (S2 Table).

### Motif or Pattern Search and Phylogenetic analysis

Motif or pattern based analysis was implemented to find out significant function for the HPs (S2 Table) to increase the power of annotation transfer. To generate multiple sequence alignment within each predicted protein families, Clustal X software was used [36]. The pattern profiles obtained as a result of multiple sequence alignment were further preceded with motif analysis using ScanProsite tool [37]. The phylogenetic tree was reconstructed using the maximum likelihood method implemented in the PhyML program (v3.1/3.0 aLRT) [38,39]. Reliability for internal branch was assessed using the bootstrapping method (100 bootstrap replicates). The program FigTree (http://tree.bio.ed.ac.uk/software/figtree/) was used for tree visualization.

### Fold Recognition

Comparing an unknown protein sequence with structure by a process of threading or fold match is another method to predict its function, known as Fold recognition [40]. The folding pattern of a protein often remains conserved during evolution and hence an unknown protein secondary structure information obtained through fold recognition may give a clue for studying their biochemical or biophysical functions [41]. Fold recognition for all the predicted GH and hydrogenase genes obtained from the above methods were done using Phyre^2^ Protein Fold Recognition Server [42]. It uses a library of known protein structures obtained from the structure databases. Iterative PSI-BLAST is used to obtain the close and remote homologs of the submitted query and a profile is constructed. The secondary structure alpha-helix (H), beta-strand (E) and coil (C) were predicted based on the best sequence alignment score. The constructed profile and secondary structures were scanned against the fold library using profile-profile algorithm and the best alignments were ranked.

### Manual genome context analysis using STRING

For all the 781 genes categorized as “hypothetical”, we identified associated ‘COGs’ groups and ‘non-supervised orthologous groups’, ‘NOGs’ using STRING (version–9.1) [43]. The confidence score calculated by STRING is based on three genomic context methods: conserved gene neighborhood, gene fusion events and significant co-occurrence of the genes across a specific subset of species. STRING quantitatively integrates interaction data from these sources for a large number of organisms, and transfers information between these organisms wherever applicable. Manual in-depth genomic context analyses were applied to the set of HPs by using methods available at the STRING database to detect conserved operon architecture or fusion events supported by co-occurrence of genes across different species.

### Genome Comparison

**Fig 1:**
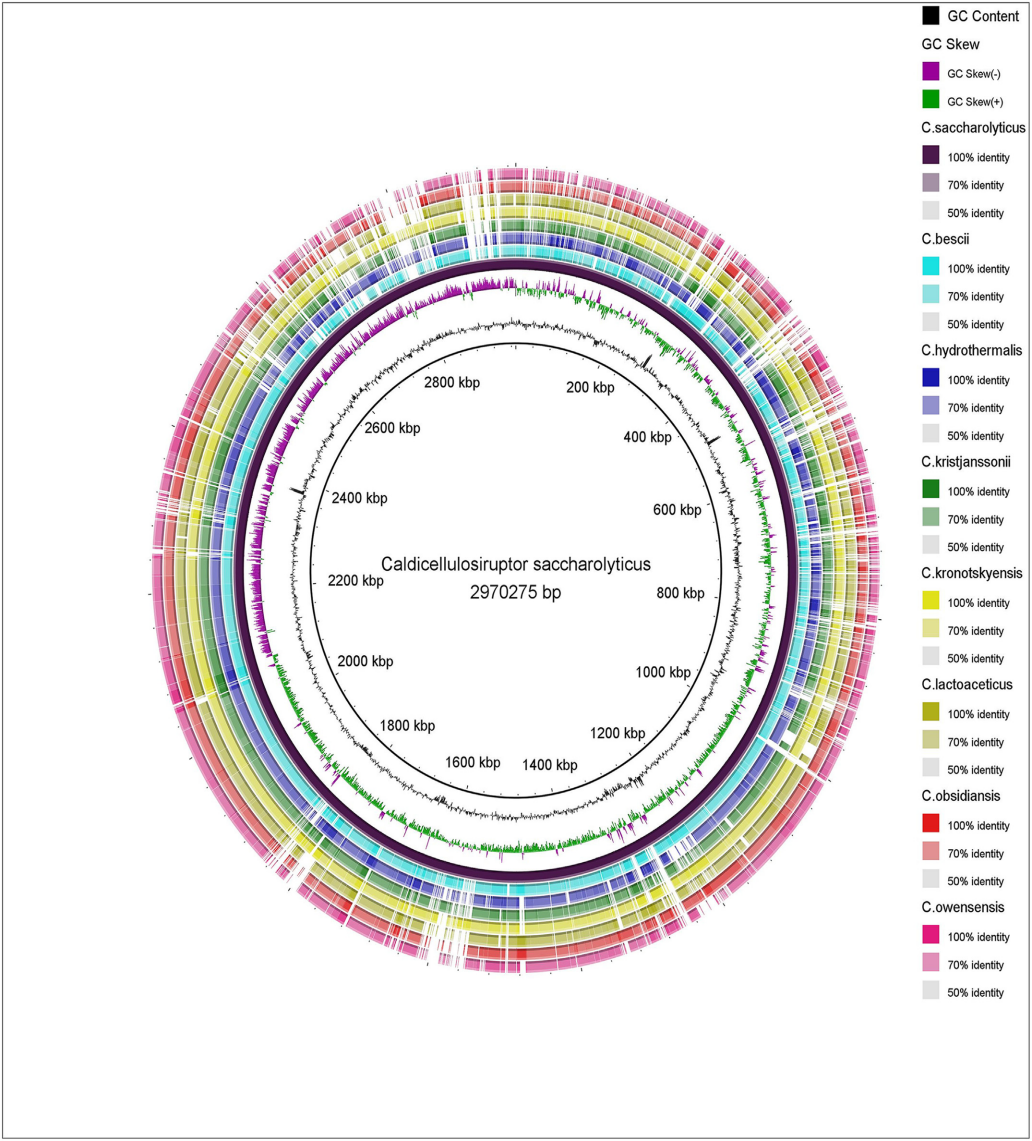
Visualization of alignment of the 8 *Caldicellulosiruptor* genomes generated using Blast Ring Image Generator (BRIG) showing (from inner to outer), % G+C, GC skew and the homology based on BLASTn. The deep purple circle represents the reference sequence, *C*. *saccharolyticus*. Outer rings show shared identity (according to BLASTn) with various other *Caldicellulosiruptor* genomes. BLASTn matches between 50% and 100% nucleotide identity are colored from lightest to darkest shade respectively, according to the graduated scale on the right of the circular BLAST image. Matches with less than 50% identity appear as blank spaces in each ring.

We assessed the homologous and syntenic genes among the members of genus *Caldicellulosiruptor*. Eight species from this genus (*C*. *saccharolyticus*, *C*. *bescii*, *C*. *hydrothermalis*, *C*. *kristjanssonii*, *C*. *kronotskyensis*, *C*. *lactoaceticus*, *C*. *owensensis* and *C*. *obsidiansis*) were compared using Gene Phyloprofile interface in MicroScope [26]. This interface is used to search for common or specific genes between a query genome and other genomes available in Prokaryotic Genome DataBase (PkGDB) or complete genome downloaded from NCBI Reference Sequence Database (RefSeq) / Whole Genome Sequencing Program (WGS) section. A default homology constraints of minLrap (>0.8) and Identity (>30%) was used to find the homologues. Sequences were plotted to check their identity using the Blast Ring Image Generator (BRIG) [44] by aligning the 7 *Caldicellulosiruptor* species genomes with *C*. *saccharolyticus* as a reference (Fig 1).

### Statistical based performance assessment

An important step towards the validation of the design of the study and their predictive ability is to perform a statistical evaluation of diagnostic accuracy [22]. ROC (Receiver Operating Characteristic) analysis is widely used method to evaluate the overall accuracy of prediction rules [45]. ROC based analysis was carried out for 1,854 known proteins and their predicted functions using various bioinformatics tools from S1 Table. An in-house Python (https://www.python.org/) script was written to classify the predicted functions by using binary numerals “0” and “1” depending on whether the case is truly positive “1” or truly negative “0” and integers (2,3,4 and 5) were used as confidence rating for each case (S3 Table). The classification results were manually cross-checked to avoid any discrepancy and submitted in format 1 form to the ‘‘ROC Analysis: Web-based Calculator for ROC Curves” [46]. This software automatically calculates a receiver operating characteristic curve by using the input data and generates output as accuracy, sensitivity, specificity and the area under the ROC curve which were further used to validate the predicted functions for HPs. The average accuracy of the used tools was found to be 91.52% (S4 Table) which indicates that the output of functional re-annotation results are reliable and can be considered for any further studies.

## Results and Discussion

### Evaluation of Genome Re-annotation

The genome of *C*. *saccharolyticus* DSM8903 consists of only 69% of proteins with known functions while the remaining 31% were classified as hypothetical or putative. So, as a step forward in improving the existing annotation of *Caldicellulosiruptor* genome, the sequences were submitted to MicroScope Platform which predicted 3,017 CDSs and these were further classified into five categories: [i] Known: proteins with known function (1,854) [ii] Hypothetical: proteins with unknown function (781) [iii] Putative: proteins with function based on a conserved motif, structural feature or limited similarities (47) [iv] New CDSs: newly predicted CDSs from MicroScope (231) and [v] Pseudogenes or gene remnants (104). Pseudogenes or gene remnants were not pursued further for re-annotation analysis.

We updated the functions of all the CDSs identified in 2008 [4] and assigned more accurate functions based on our manual analysis results using a wide range of bioinformatics tools (PSI-BLAST, BLASTO, Pfam, InterProScan and ANNIE). For example, Csac_0067 was predicted as Zeta toxin family protein in the original annotation. In our re-annotation studies, we have attempted to give detailed information about some of the known proteins with PMID for example, Zeta toxin function is to inhibit cell wall biosynthesis and it may act as a bactericide in nature (PMID: 22295078). No conflicting annotations were found in the case of the known genes.

**Fig 2:**
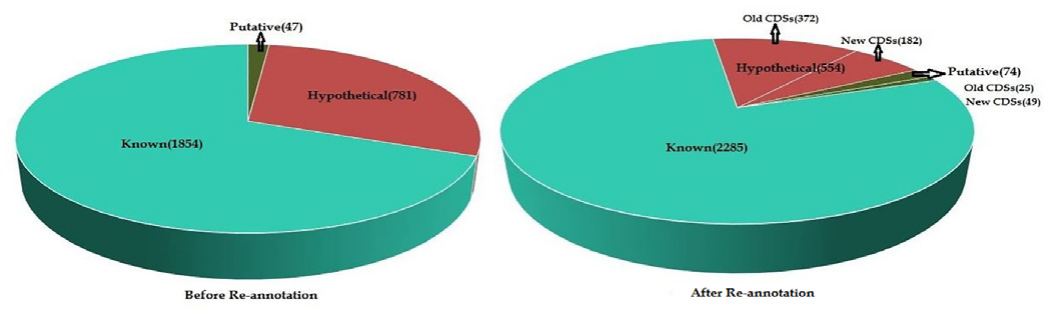
Statistics of Known, Hypothetical and Putative genes before and after re-annotation. Before re-annotation category include: Known (1854), Hypothetical (781) and Putative (47). After re-annotation category include: Known (2285), Hypothetical: old CDSs (372) + new CDSs (182) and putative: old CDSs (25) + new CDSs (49).

In the original annotation 781 and 47 CDSs were found to be hypothetical and putative respectively. Thus, using the above mentioned approach 385 and 24 HPs were predicted with well defined functions and putative functions respectively, among which 23 HPs were characterized with complete confidence that is for each of the sequence, function was predicted from all five tools. We successfully assigned a proposed function with high confidence to 46 out of 47 CDSs previously annotated as putative. Subsequently, the number of genes with known functions increased from 1,854 to 2,285 and that with putative functions decreased from 47 to 25 (Fig 2). Here, 25 sequences with putative functions include 24 HPs whose functions are predicted as putative after re-annotation and 1 out of 47 previously annotated as putative.

ABC-transporters facilitate the transport of soluble sugar substrates like mono-, di-, oligosaccharides across the membrane of *C*. *saccharolyticus* at the expense of ATP [12]. We have successfully predicted 11 ABC Transporter genes from our re-annotation effort with high confidence level (i.e. 3 out of 5 tools predicted the same function for the CDS). Major facilitator superfamily (MFS) is another class of membrane transport protein that facilitates movement of small solutes across cell membrane and we predicted Csac_1704 as major facilitator superfamily protein, but it is likely that carbohydrate utilization in this organism proceeds mainly through ABC transporters [4]. *C*. *saccharolyticus* chromosome like many other prokaryotes contains clustered regularly interspaced short palindromic repeats (CRISPR). They are DNA repeats that are separated by highly variable intervening sequences (spacers) and accompanied by CRISPR-associated (CAS) genes [4]. The function proposed for CRISPR and CAS protein is to provide resistance to bacteriophages [47]. We identified 7 HPs Csac_0050, Csac_0059, Csac_0487, Csac_0489, Csac_0313, Csac_0488, Csac_0312, to function as CRISPR-associated protein. In addition to this, we found 28 transposase that are responsible for high level of plasticity in this organism, 6 transcriptional regulators, 2 Regulatory protein recX and yycI, 1 DNA-binding regulatory protein (YebC/PmpR family) and 1 Phosphate transport regulator protein. All the information about the re-annotation of the known and unknown genes using different tools are available in S1 and S2 Tables respectively. All information about the re-annotated CDSs available in S2 Table is currently available on the MicroScope platform (https://www.genoscope.cns.fr/agc/microscope/mage/viewer.php?S_id=2771) and in tabulated flat file format at the following URL: https://www.genoscope.cns.fr/agc/microscope/search/export.php?format=csv&part=all&S_id=2771

The genomic distribution of proteins in COG categories before and after re-annotation is compared in Table 1. 2,556 CDSs were classified into at least one COG group after re-annotation. Table 1 shows a major difference in the number of proteins reported in each of the COG functional categories before and after re-annotation, e.g. the number of genes reported in Carbohydrate transport and metabolism in the original annotation was 213 which after re-annotation increased to 237. Only Coenzyme transport and metabolism showed a decrease in the number of protein sequences, that is, from 101 to 95. 357 protein encoding genes could not be classified in any of the COG functional categories. Two COG functional categories exhibiting the greatest change in their number of protein encoding genes are General function prediction only (R) category with 316 and the Replication, recombination and repair (L) category with 279 numbers of genes. We successfully classified 102 out of 781 HPs into at least one COG functional category.

**Table 1:** Functional categories of Coding Sequences in the genome of *Caldicellulosiruptor saccharolyticus* before and after re-annotation.

COG category	COG functional category	Number of CDSs in each category* (2008) [4]	Number of CDSs in each category*: After re-annotation, (2015)(X/Y)^#^
INFORMATION STORAGE AND PROCESSING			
**J**	Translation, ribosomal structure and biogenesis	147	157
**K**	Transcription	134	158/2
**L**	Replication, recombination and repair	222	273/6
**B**	Chromatin structure and dynamics	2	2
METABOLISM			
**E**	Amino acid transport and metabolism	166	200/1
**F**	Nucleotide transport and metabolism	56	57
**G**	Carbohydrate transport and metabolism	213	236/1
**H**	Coenzyme transport and metabolism	101	95
**C**	Energy production and conversion	111	115
**P**	Inorganic ion transport and metabolism	73	100
**Q**	Secondary metabolites biosynthesis, transport and catabolism	14	24
**I**	Lipid transport and metabolism	34	39
CELLULAR PROCESSES AND SIGNALING			
**M**	Cell wall/membrane/envelope biogenesis	107	122/2
**D**	Cell cycle control, cell division, chromosome partitioning	35	57
**T**	Signal transduction mechanisms	125	126/3
**U**	Intracellular trafficking, secretion, and vesicular transport	42	55
**V**	Defense mechanisms	48	56/2
**Z**	Cytoskeleton	3	3
**N**	Cell motility	71	81
**O**	Posttranslational modification, protein turnover, chaperones	59	67/1
POORLY CHARACTERIZED			
**R**	General function prediction only	228	315/1
**S**	Function unknown	177	199

*Number of protein-encoding genes in each category without pseudogenes.

^#^ (X/Y) = > X: value belonging to the old CDSs / Y: value belonging to the new CDSs.

Currently large numbers of complete genome sequences are available in the public databases and it is necessary to identify genes involved in the maintenance of the cell. Gil et al. [34] reconstructed the minimal metabolic machinery needed to sustain life, based on the data available from several experimental approaches to define all the essential genes in some completely sequenced bacterial genomes. The minimal genome set is composed of 206 protein coding genes that include well conserved housekeeping genes for basic metabolism and macromolecular synthesis in a bacterial genome. The list of minimal gene sets for *C*. *saccharolyticus* was generated from the MicroScope platform (S5 Table). Among the list of essential genes, 7 of them Csac_2571, Csac_1262, Csac_0970, Csac_1701, Csac_2220, Csac_1839 and Csac_2091, were found to be hypothetical and the re-annotation study predicted their function as O-antigen polymerase, histone family DNA-binding protein, uracil-DNA glycosylase, glycyl-tRNA synthase subunit, dCTP deaminase, metalloprotease ybeY and aminopeptidase pepA respectively. Also, we found that 18 essential genes described in Gil et al. [34] were not found. From our re-annotation results, we suggest that the hypothetical gene Csac_2372 function as the missing essential gene Ndk-nucleoside diphosphate kinase. Ndk plays an important role in bacterial growth, signal transduction and pathogenicity [48].

### Hydrolytic Capacity and Complex Biomass Decomposition

*C*. *saccharolyticus* does not make use of Cellulosome-like structures for the decomposition of complex plant polysaccharides, as reported in some of the *Clostridium* species [12,49], but its genome encodes a wide variety of endo- and exo-glycoside hydrolases capable of complex biomass degradation like starch, pullulan and cellulose, but also xylan and hetero-polysaccharides like hemicelluloses and pectin [12,50,51,52,53]. Using different bioinformatics tools, based on sequence similarity 7 HPs were predicted as GH in *C*. *saccharolyticus* (Table 2) that are proposed to be involved in complex biomass degradation. We identified Csac_0590 and Csac_0661 as S-layer domain-containing protein that is known to play a significant role in cell substrate interactions. Interactions between whole cells and a substrate have also been observed for *C*. *saccharolyticus* in addition to substrate and glycoside hydrolase interactions [12]. S-layer domain-containing proteins contain both GH domains and non-catalytic carbohydrate binding domains which are utilized in lignocellulose degradation by both recruiting and degrading complex biomass via cell substrate interactions.

**Table 2:** List of Predicted Glycoside hydrolase, S-layer domain containing proteins, hydrogenase, and iron-sulfur clusters (with confidence level) using sequence similarity based approach.

Label	RefSeqID	CG Cont-ent	Annotated Function	Re-annotated Function	Confidence level
**Csac_0437**	**YP_001179266.1**	**0.39**	**hypothetical protein**	**Beta-xylosidase, family 43 glycosyl hydrolase**	**5/5**
**Csac_0884**	**YP_001179692.1**	**0.3149**	**hypothetical protein**	**glycosyl hydrolase family 98 proteins**	**3/5**
**Csac_2528**	**YP_001181294.1**	**0.3306**	**hypothetical protein**	**Cellulase (glycosyl hydrolase family 5)**	**4/5**
**Csac_2723**	**YP_001181482.1**	**0.3248**	**hypothetical protein**	**beta-galactosidase**	**4/5**
**Csac_1016**	**YP_001179821.1**	**0.3351**	**hypothetical protein**	**alpha/beta superfamily hydrolase**	**4/5**
**Csac_0424**	**YP_001179254.1**	**0.3851**	**hypothetical protein**	**glycoside hydrolase family 2, sugar binding protein**	**2/5**
**Csac_0206**	**YP_001179045.1**	**0.3915**	**hypothetical protein**	**beta-galactosidase/beta-glucuronidase**	**2/5**
**Csac_0590**	**YP_001179407.1**	**0.3499**	**hypothetical protein**	**S-layer domain-containing protein**	**3/5**
**Csac_0661**	**YP_001179472.1**	**0.3772**	**hypothetical protein**	**S-layer domain-containing protein**	**3/5**
**Csac_1862**	**YP_001180638.1**	**0.3487**	**hypothetical protein**	**NAD(P)-dependent iron-only hydrogenase iron-sulfur protein**	**5/5**
**Csac_0732**	**YP_001179542.1**	**0.2874**	**hypothetical protein**	**FMN-binding domain-containing protein**	**4/5**
**Csac_1294**	**YP_001180087.1**	**0.3626**	**hypothetical protein**	**4Fe-4S ferredoxin, iron-sulfur binding domain**	**4/5**
**Csac_0668**	**YP_001179479.1**	**0.3832**	**hypothetical protein**	**(2Fe-2S)-binding domain-containing protein**	**2/5**

9 hypothetical sequences predicted as GH and S-layer domain-containing proteins were further analyzed for conserved motif or pattern identification, structural and phylogenetic investigation, since functional prediction based on sequence similarity alone cannot be evident enough to assign its accurate function. The conserved motifs for the predicted GH were identified using Clustal X software (S1 Fig). The conserved signature pattern identified for Csac_0437 was [HQ]-E-G-P-N-V-F position 189 to 195 from the region 7–301 containing a five-bladed beta-propellar domain found in some GH, and this motif was found specific to Glycoside hydrolase, family 43 when searched in ScanProsite tool. Csac_0424 was found to contain a galactose-binding domain in the region 862–947. The structure of the galactose-binding domain-like members consists of a beta-sandwich, in which the strands making up the sheets exhibit a jellyroll fold. The signature pattern was identified from this region as D-I-T-[RK]-[SD]-G-Y-P-F-Y-V-G-[ST] and the presence of this motif reveals its specificity to Glycoside hydrolase family 2, sugar binding protein. No significant results were obtained for 7 out of 9 HPs predicted as GH and S-layer domain-containing proteins based on sequence similarity.

Phylogenetic analysis of the predicted HPs Csac_0437 and Csac_0424 has been performed with 8 and 13 sequences respectively from closely related species. The phylogenetic tree was used to cluster closely related members of the GH family proteins. As a result, HP Csac_0437, which was re-annotated as Glycoside hydrolase, family 43, exhibit that it is a part of cluster containing known Glycoside hydrolase family proteins (WP_013429358.1, WP_013412975.1, WP_013402237.1) (S2 Fig). Similarly, Csac_0424 forms a cluster with known GH family proteins from other organisms (S3 Fig). Thus, Csac_0437 and Csac_0424 and its close homologs are likely to share a similar function.

Fold recognition studies of a HP were performed to predict the functional three-dimensional folds within the protein. The hypothetical sequence Csac_0437 was predicted to have Hydrolase fold with a confidence of 100%, with confidence greater than 90% one can be confident that the core of protein is modeled at high accuracy and the protein adopts the fold. The percentage identity between the sequence and the template 2EXI was found to be 16%. It is well evident from literature that the sequence identity should be above 30–40% for highly accurate models, but if the sequence identity are even less, models with high confidence can still be useful. The RMSD (root mean square deviation) value was obtained as 2.15Å (optimum RMSD value ranges from 2-4Å [42]). Similarly, Csac_0424 also has Hydrolase fold with a high confidence of 99.9% and shows structural similarity of 12% with the template 3TTS. The RMSD value was found to be 2.40Ǻ.

### Fermentation Products leading to hydrogen production

Embden-Meyerhof pathway is the main route for glycolysis in *C*. *saccharolyticus* and has been experimentally validated by the Nuclear magnetic resonance (NMR) analysis of the end-products of the organism grown on ^13^C-labeled glucose [54]. The complete catabolization of each sugar substrate to glyceraldehydes 3-phosphate (GAP) and then finally to pyruvate leads to the production of reduced electron carrier nicotinamide adenine dinucleotide (NADH). Finally, the formation of acetyl-CoA from pyruvate results in the formation of reduced ferredoxin (Fd_red_). NADH and Fd_red_ are subsequently used by hydrogenases to generate H_2_ [4]. Hydrogenase/dehydrogenase types of enzymes play a significant role in biohydrogen production [1,55,56,57].

In this re-annotation work, we predicted 2 Fe-S oxidoreductases (Csac_0899 and Csac_2647), 1 Pyruvate/2-oxoglutarate dehydrogenase (Csac_0872), 3 methanol dehydrogenases (Csac_2587, Csac_2589, Csac_0034), 1 NAD(P)-dependent iron-only hydrogenase iron-sulfur protein (Csac_1862), 1 FMN-binding domain-containing protein/4Fe-4S binding domain (Csac_0732), 1 4Fe-4S binding domain containing proteins (Csac_1294), 1 BFD domain-containing protein (2Fe-2S)-binding domain-containing protein (Csac_0668) and 1 multi-copper polyphenol oxidoreductase laccase (Csac_2035). Iron-sulfur clusters are found in variety of metalloproteins (such as ferredoxins, as well as NADH dehydrogenase and hydrogenases) and are best known for their role in the oxidation-reduction reactions [58]. Csac_0732, Csac_1862, Csac_1294 and Csac_0668, were assumed to be directly involved in H_2_ production and are taken further for conserved motif or pattern identification, structural and phylogenetic studies. The conserved signature pattern for Csac_0732 was identified as G-R-[FW]-F-C-G-W-[VIM]-C-A-F-G (S4 Fig) which indicates that this region is highly rich in Gly and represents FMN-binding domain proteins [59]. For Csac_1862 the presence of G-C-I-G-[MVL]-C-[KR] signature pattern revealed that it has GxGxxGxxxG [59] motif specific to NAD(P)-dependent iron-only hydrogenase. The sequence Csac_1294 possesses the pattern C-I-R-C-[YF]-C-C-[HQ]-E-L-C-P which revealed that it contains the fingerprint motif CXXCXXC [59] specific to 4Fe-4S cluster. Likewise, Csac_0668 has the conserved motif pattern C-[PS]-[IV]-C that indicates the presence of CXXC motif specific to (2Fe-2S)-binding domain-containing protein [59].

In addition, Csac_0732 illustrated that it forms a cluster with known FMN-binding proteins YP_004003231.1, YP_003841245.1, YP_003991656.1 and YP_004023144.1 (S5 Fig). Similarly, Csac_1862 clustered with closely related members containing the hypothetical gene Csac_1862 and ferredoxin genes WP_013403318.1, WP_013412030.1, WP_013430367.1 and WP_013290551.1 (S6 Fig). Furthermore, Csac_1294 and Csac_0668 re-annotated as 4Fe-4S binding domain containing protein and 2Fe-2S binding protein are part of the clusters containing known 4Fe-4S binding domain proteins (S7 Fig) and 2Fe-2S binding proteins respectively (S8 Fig).

Fold recognition results for the HP Csac_0732 showed that it has oxidoreductase fold with a confidence of 23.0%, and whose predicted backbone RMSD value was obtained as 1.78Å. In addition to this, the hypothetical sequence Csac_1862 was predicted to have Thioredoxin fold with a confidence of 99.9% with the Template 1F37. The RMSD value was obtained as 2.7Å. The predicted 4Fe-4S binding domain containing protein Csac_1294 contains an oxidoreductase fold with a confidence of 99.6% and a RMSD value of 1.93Å. The template was 2C3Y which codes for pyruvate-ferredoxin oxidoreductase. Csac_0668 was also found to have oxidoreductase fold with a confidence of 98.9% and a RMSD value of 2.86Å.

### Functional clues for Hypothetical proteins based on genomic context analysis

**Fig 3:**
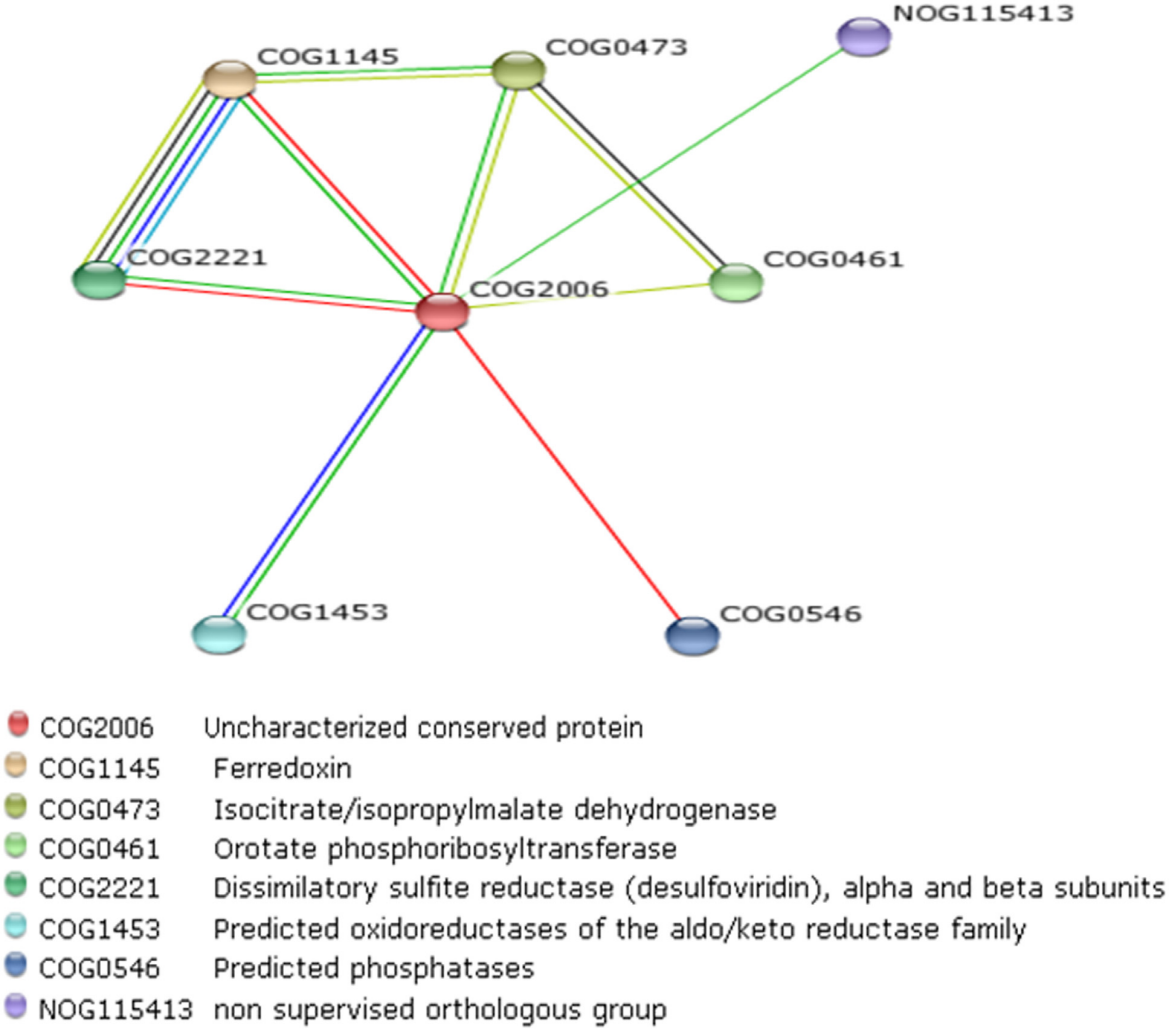
Figure showing a network of predicted associations for a particular group of proteins (related to COG2006 (containing the hypothetical protein Csac_1294 (red)) which is predicted to be involved in valine, leucine and isoleucine biosynthesis pathway and oxidation-reduction process. The network edges represent the predicted functional associations. Any edge may be drawn with differently coloured lines: a red line indicates the presence of fusion evidence; a green line—neighborhood evidence; a blue line—Co-occurrence evidence, a black line—Co-expression evidence, a yellow line—text mining evidence, and a light blue line indicates database evidence.

Using a confidence score greater than 0.4 (medium confidence), genomic context networks for the set of 781 HPs was built to visualize their operon architectures as well as their fusion events and co-occurrence of genes across different species. We found the functional associations for these uncharacterized proteins to a broad variety of cellular activities like transcription and translation, metabolic processes, transporter activity, DNA recombination and repair. The HP Csac_0732 is a member of COG0348, which is significantly linked to several genes involved in oxidation reduction process. In firmicutes, the considered hypothetical gene was found to be a part of an operon which contains ferrodoxin genes [4Fe-4S cluster], participating in electron transfer, substrate binding/activation, iron/sulfur storage, regulation of gene expression, and enzyme activity. Our analysis proposed a clear involvement of this HP in oxidation reduction process. Similarly, HP Csac_1294 a member of uncharacterized conserved protein COG2006 was found to be located in an operon which contain several genes with 3-isopropylmalate dehydrogenase as function and iron sulfur cluster binding proteins (Fig 3). 3-isopropylmalate dehydrogenase catalyzes the oxidation of 3-carboxy-2-hydroxy-4-methylpentanoate (3-isopropylmalate) to 3-carboxy-4-methyl-2-oxopentanoate. Csac_1294 can be predicted to be involved in valine, leucine and isoleucine biosynthesis pathway and oxidation-reduction process. Finally, for 196 COGs and 53 NOGs we were able to predict a cellular role, a connected pathway or a complex (S6 Table).

### Revising the number of genes in the genome

*C*. *saccharolyticus* genome in NCBI-Microbial Genome Database consists of 2,682 CDSs. Following the re-annotation, we have now included 231 new CDSs whereas no changes have been detected in the number of RNA molecules. The new CDSs use “CALS8” as the preceding identifier followed by a four-digit number both separated by an underscore (e.g. CALS8_0355, CALS8_0367, CALS8_0578). Each of the newly predicted CDS was subjected to a wide range of bioinformatics tools PSI-BLAST, BLASTO, Pfam, ANNIE and InterProScan and we assigned putative functions to 49 of such sequences. We added ‘putative’ prior to the product annotation since the proposed functions were based on presence of conserved amino acid motif, structural feature or limited homology (S7 Table). Functions of 8 out of 49 CDSs were predicted with complete confidence (i.e. all 5 tools predicted the same function). 3 of them share homology with CRISPR-associated (CAS) genes that provides resistance to bacteriophages. 1 coding sequence was predicted as putative Transposase, Mutator family which is important for an organism’s plasticity. 2 of the newly predicted CDSs function as putative diguanylate cyclase and is expected to participate in the formation of cyclic di-3’,5’-guanylate (cyclic-di-GMP) which is further involved in bacterial biofilm formation and persistence. 2 out of 8 sequences were predicted as putative IstB-like ATP binding protein and putative MerR family regulatory protein. IstB-like ATP binding proteins have not been characterized functionally, but they may be involved in transposition while MerR family regulatory protein has sequence-specific DNA binding transcription factor activity. Finally, after re-annotation the new CDSs were classified into genes with putative functions (49) and genes without functions or hypothetical (182) (Fig 2). 19 out of 231 new CDSs were classified into one of the COG functional categories and are shown in Table 3.

**Table 3:** Functional categories of newly predicted Coding Sequences in the genome of *Caldicellulosiruptor saccharolyticus*.

Labels	COG class ID	COG functional category	COG process
CALS8_0014,CALS8_0429,CALS8_0430,	T	Signal transduction mechanisms	CELLULAR PROCESSES AND SIGNALING
CALS8_1121,CALS8_2818	V	Defense mechanisms	CELLULAR PROCESSES AND SIGNALING
CALS8_1664,CALS8_2385	M	Cell wall/membrane/ envelope biogenesis	CELLULAR PROCESSES AND SIGNALING
CALS8_2734	O	Posttranslational modification, protein turnover, chaperones	CELLULAR PROCESSES AND SIGNALING
CALS8_0355,CALS8_0358,CALS8_0367,CALS8_0544,CALS8_0639,CALS8_2622	L	Replication, recombination and repair	INFORMATION STORAGE AND PROCESSING
CALS8_0578,CALS8_1233	K	Transcription	INFORMATION STORAGE AND PROCESSING
CALS8_1211	G	Carbohydrate transport and metabolism	METABOLISM
CALS8_2733	E	Amino acid transport and metabolism	METABOLISM
CALS8_2384	R	General function prediction only	POORLY CHARACTERIZED

### Diversity of *Caldicellulosiruptor* species

Comparative analyses of the gene content of all eight species of the genus *Caldicellulosiruptor* were carried out to get core genome, the set of orthologous genes shared by the eight species. The candidate core genome contains 1,787 genes and 321 genes were found specific to *C*. *saccharolyticus*. In the case of whole genome pairwise comparison between *C*. *saccharolyticus and* other 7 species *(C*. *bescii*, *C*. *hydrothermalis*, *C*. *kristjanssonii*, *C*. *kronotskyensis*, *C*. *lactoaceticus*, *C*. *owensensis* and *C*. *obsidiansis)*, we found that *C*. *bescii* shared the highest number of homologous genes (2,420) with *C*. *saccharolyticus* indicating them to be evolutionary more close compared to others.

Chromosomal synteny analysis is an important step in genome comparison to reveal genomic evolution of related species. Shared synteny describes genomic fragments from different species that originated from an identical ancestor. Syntenic genes are orthologs located in syntenic fragments for which function annotation information can be shared with high confidence [60]. Chromosomal synteny based whole genome comparison showed the presence of 1,627 genes of *C*. *saccharolyticus* with syntenic counterparts in all the other 7 species. 2 HPs predicted as Fe-S clusters and 6 HPs and new CDSs predicted as CRISPR-associated genes were found syntenous with known genes from other *Caldicellulosiruptor* species which helped us to improve annotations at these loci (S8 Table). These annotations provide a starting point for further investigation of these proteins in *Caldicellulosiruptor*.

Comparative genomics approach was used [61,62] to examine the conservation of essential genes identified in *C*. *saccharolyticus* (S5 Table) among *Caldicellulosiruptor* species. The vast majority of essential genes (171 of 205) had orthologs in all of the other 7 *Caldicellulosiruptor* genomes. Thus, in agreement with expectation, most of the genes we identified as essential were highly conserved. The conserved essential genes were distributed in specific categories based on KEGG pathway maps [63] and examined. The categories with highest percentage of essential genes were translation (44%), Energetic and Intermediary Metabolism (17%), Protein processing, folding, and secretion (7%), and DNA metabolism (7%). The essential genes were clustered in many pathways: (i) glycolysis, (ii) pentose phosphate pathway, (PPP), (iii) lipid metabolism, (iv) nucleotide biosynthesis, (v) biosynthesis of cofactors and nucleotides, (vi) cell division and transport, (vii) DNA replication, (viii) transcription and translation, (ix) oxidative phosphorylation.

## Conclusions

In the presence of the whole genome sequences of an organism, the incomplete functional annotation of sequences hampers the ability to exploit these data for any application purposes. So, with an aim of improving annotation for *C*. *saccharolyticus*, all the CDSs were manually reviewed and assigned with a more accurate and appropriate function using a number of bioinformatics tools. A homology based approach that included similarity search, motif or pattern search, phylogenetic analysis and fold prediction was applied to validate the functions. In addition 231 new CDSs were identified and 49 of them were assigned with putative functions using various bioinformatics tools. Finally, after re-annotation 2,285 and 74 proteins were assigned with well defined functions and putative functions respectively. To complement the homology-based methods, genomic context-based method for function prediction was also used and we successfully predicted functional association for 249 out of 781 HPs. Functional analysis of several HPs such as GH and hydrogenases or Fe-S clusters, will further help us to enhance the decomposition of substrates by hydrolyzing the glycosidic bonds of α- and β-glucans to form oligo- or mono-saccharides, and the formation of H_2_ by using both types of reduced electron carriers (NADH and Fd_red_) respectively. The list of essential genes that includes the smallest number of genes essential for the survival of the organism under the most favorable conditions, were identified by performing homology search between each gene of the organism of interest and the set of 206 essential gene reported by Gil et al. [34]. In our essential gene comparison, we found significant conservation of essential genes identified in *C*. *saccharolyticus* among members of genus *Caldicellulosiruptor*. Hence, this whole genome re-annotation approach will further help to better understand the metabolic pathway network in *C*. *saccharolyticus* and thus, in turn, will facilitate the potential of H_2_ production and make it economically suitable for commercial purposes. In future, the predicted functions of some of the important HPs related to biomass degradation and H_2_ production by using bioinformatics approach, will be experimentally validated by using standard wet lab techniques.

## Supporting Information

S1 Fig Multiple sequence alignment of predicted Glycoside Hydrolase (Csac_0437) using Clustal X software. The corresponding NCBI RefSeq accession numbers and organisms are listed below: *C*. *saccharolyticus* (Csac_0437); *C*. *kronotskyensis*, WP_013429358.1; *C*. *hydrothermalis*, WP_013402237.1; *C*. *owensensis*, WP_013412975.1; *Paenibacillus mucilaginosus* 3016, YP_005315616.1; *Clostridium phytofermentans*, WP_012199080; *Paenibacillus* sp. JDR-2, YP_003012324.1; *Clostridium sp*. D5, WP_009001969.1.
(TIF)

S2 Fig Phylogenetic tree result for Csac_0437. The corresponding NCBI RefSeq accession numbers and organisms list are same as that of S1 Fig.
(TIF)

S3 Fig Phylogenetic tree result for Csac_0424. The corresponding NCBI RefSeq accession numbers and organisms taken for phylogenetic studies are as follows: *C*. *saccharolyticus* (Csac_0424); *C*. *hydrothermalis*, WP_013402227.1; *C*. *bescii*, WP_015908894.1; *C*. *kronotskyensis*, WP_013429348.1; *C*. *kristjanssonii*, WP_013431541.1; *C*. *owensensis*, WP_013412985.1; *C*. *obsidiansis*, WP_013291434.1; *Thermotoga sp*. EMP, WP_008195720.1; *Thermotoga sp*. RQ2, YP_001739759.1; *Thermotoga petrophila* RKU-1, YP_001245251.1; *Thermotoga naphthophila* RKU-10, YP_003347175.1; *Thermotoga maritima* MSB8, YP_008991830.1; *Thermotoga neapolitana* DSM 4359, YP_002535037.1.
(TIF)

S4 Fig Multiple sequence alignment of predicted FMN-binding domain-containing protein/4Fe-4S binding domain (Csac_0732) using Clustal X software. The corresponding NCBI RefSeq accession numbers and organisms are *C*. *saccharolyticus* (Csac_0732); *C*. *obsidiansis* OB47, YP_003841245.1; *C*. *kronotskyensis* 2002, YP_004023144.1; *C*. *hydrothermalis* 108, YP_003991656.1; *C*. *owensensis* OL, YP_004003231.1; *Clostridium carboxidivorans*, WP_007059726.1; *Clostridium saccharoperbutylacetonicum* N1-4(HMT), YP_007453869.1.
(TIF)

S5 Fig Phylogenetic tree result for Csac_0732. The corresponding NCBI RefSeq accession numbers and organisms taken for phylogenetic study of this hypothetical protein is same as that of S4 Fig.
(TIF)

S6 Fig Phylogenetic tree result for Csac_1862. The corresponding NCBI RefSeq accession numbers and organisms taken for phylogenetic study are as follows: *C*. *saccharolyticus* (Csac_1862); *Caldicellulosiruptor* (multispecies), WP_013430367.1 and WP_013403318.1; *C*. *obsidiansis*, WP_013290551.1; *C*. *owensensis*, WP_013412030.1; *Clostridium clariflavum* DSM 19732, AEV67963.1; *Clostridium thermocellum* BC1, CDG35018.1; *Clostridium thermocellum* ATCC 27405, YP_001036771.1; *Thermoanaerobacterium saccharolyticum* JW/SL-YS485, ACA51659.1; *Thermoanaerobacterium xylanolyticum*, WP_013788349.1; *Thermoanaerobacterium thermosaccharolyticum* M0795, YP_007298419.1; *Thermoanaerobacter*, WP_012994911.1.
(TIF)

S7 Fig Phylogenetic tree result of Csac_1294. The corresponding NCBI RefSeq accession numbers and organisms are: *C*. *saccharolyticus* (Csac_1294); *Peptoclostridium difficile*, WP_021384294.1; *Clostridium difficile* P50, EQJ92280.1; *Clostridium bifermentans*, WP_021429406.1; *Thermoanaerobacterium xylanolyticum*, WP_013788320.1; *Desulfitobacterium dichloroeliminans*, WP_015261149.1; *Thermoanaerobacterium saccharolyticum*, WP_014758945.1; *Thermoanaerobacterium thermosaccharolyticum*, WP_013297635.1; *Desulfitobacterium dehalogenans*, WP_014792634.1.
(TIF)

S8 Fig Phylogenetic tree result of Csac_0668. The corresponding NCBI RefSeq accession numbers and organisms are: *C*. *saccharolyticus* (Csac_0668); *C*. *hydrothermalis*, WP_013403070.1; *C*. *bescii*, WP_015908008.1; *C*. *owensensis*, WP_013412264.1; *C*. *obsidiansis*, WP_013290289.1; *C*. *kronotskyensis*, WP_013430123.1; *Lachnoclostridium phytofermentans*, WP_012199821.1; *Clostridium sp*. *BNL1100*, WP_014315034.1.
(TIF)

S1 Table Re-annotation results of known proteins as well as proteins with putative functions of *Caldicellulosiruptor saccharolyticus* using 5 different bioinformatics tools based on sequence similarity. (XLSX)

S2 Table List of re-annotated function (with confidence level) of 781 Hypothetical Proteins from *Caldicellulosiruptor saccharolyticus* using PSI-BLAST, BLASTO, Pfam, ANNIE, InterProScan, Clustal X (motif search), ScanProsite and Sub-cellular localization using PSORTb. (XLSX)

S3 Table List of functionally re-annotated 1,854 proteins with known functions from *Caldicellulosiruptor saccharolyticus* using PSI-BLAST, BLASTO, Pfam, ANNIE, InterProScan for ROC analysis. (XLSX)

S4 Table List of accuracy, sensitivity, specificity and Empiric ROC area of five bioinformatics tools used for predicting functions of *Caldicellulosiruptor saccharolyticus* obtained after ROC analysis. (XLSX)

S5 Table Minimal Gene Set of *Caldicellulosiruptor saccharolyticus* DSM 8903. (XLSX)

S6 Table Hypothetical Proteins and their predicted putative functions and pathways. (XLSX)

S7 Table Function prediction of the new CDSs using various bioinformatics tools. (DOCX)

S8 Table Synteny conservation of predicted Fe-S clusters and CRISPR-associated protein genes across the members of genus *Caldicellulosiruptor*. (DOCX)
