# Supplementary figures and images for: Genome Wide Re-Annotation of Caldicellulosiruptor saccharolyticus with New Insights into Genes Involved in Biomass Degradation and Hydrogen Production
Source: PLoS One. 2015 Jul 21;10(7):e0133183. doi: 10.1371/journal.pone.0133183 (PMC4510573; doi:10.1371/journal.pone.0133183)

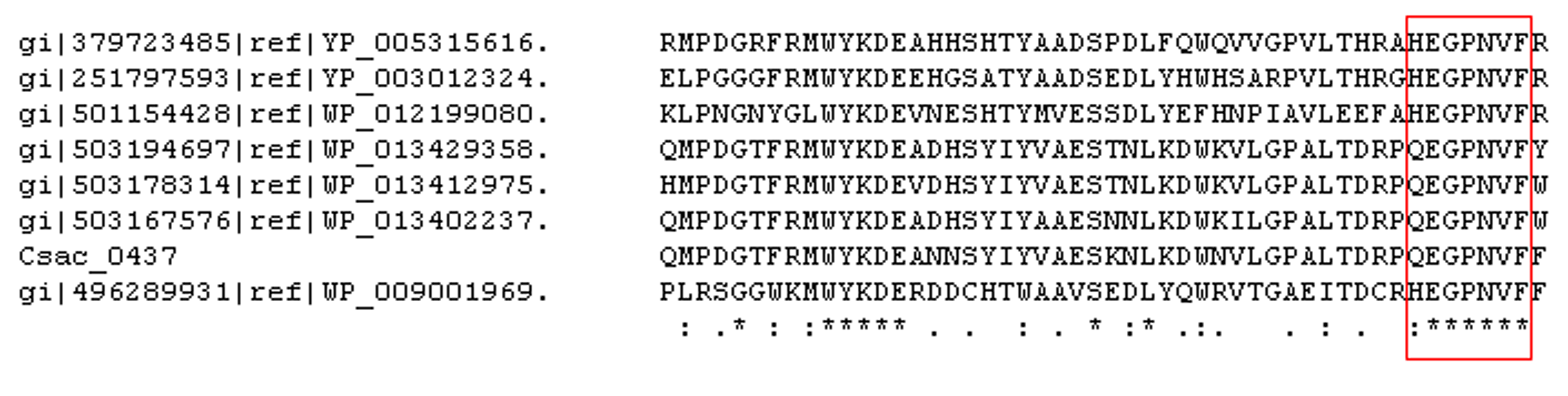

Supplement: S1 Fig — The corresponding NCBI RefSeq accession numbers and organisms are listed below: C. saccharolyticus (Csac_0437); C. kronotskyensis, WP_013429358.1; C. hydrothermalis, WP_013402237.1; C. owensensis, WP_013412975.1; Paenibacillus mucilaginosus 3016, YP_005315616.1; Clostridium phytofermentans, WP_012199080; Paenibacillus sp. JDR-2, YP_003012324.1; Clostridium sp. D5, WP_009001969.1. (TIF) [file pone.0133183.s001.tif]

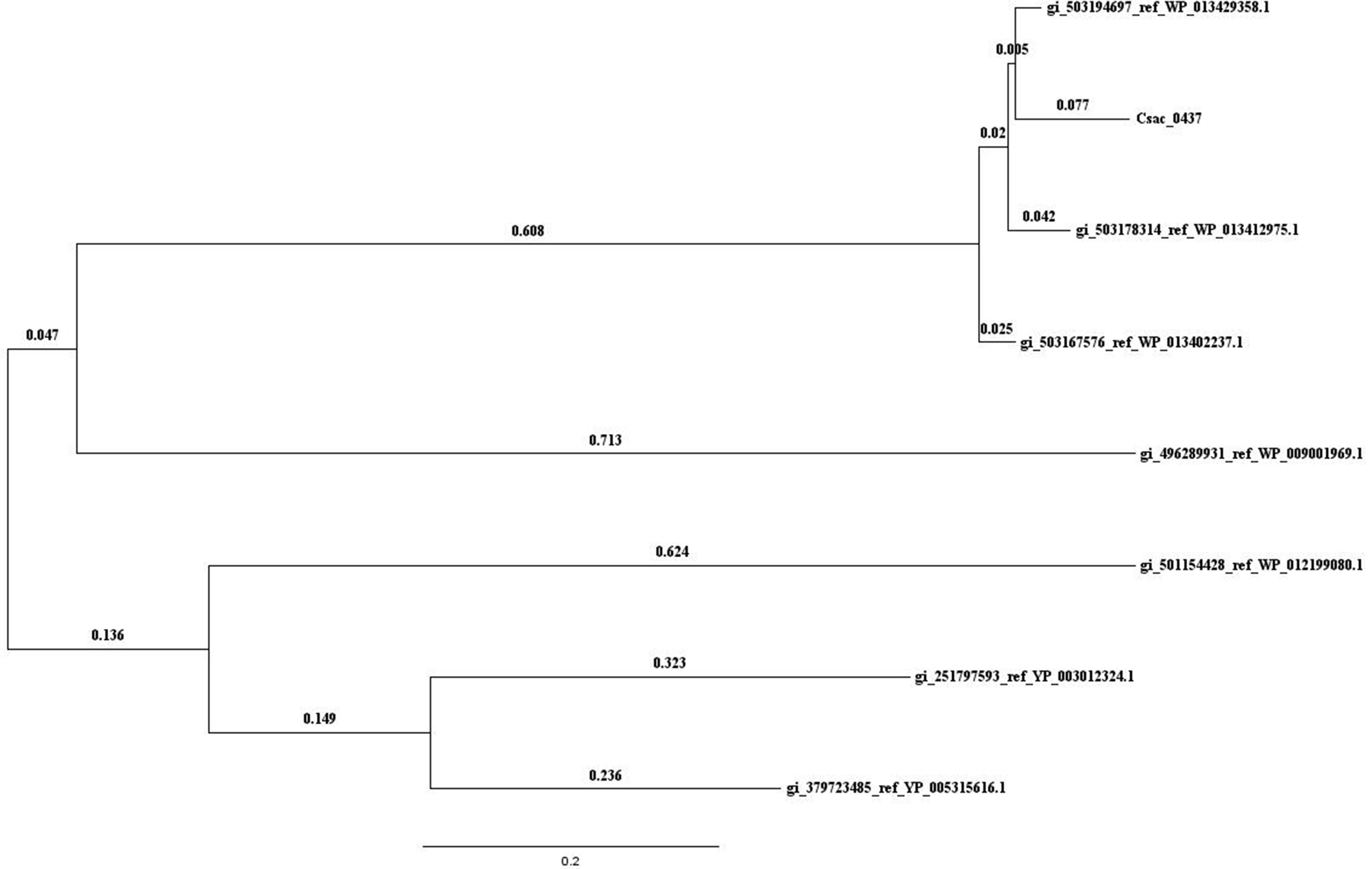

Supplement: S2 Fig — The corresponding NCBI RefSeq accession numbers and organisms list are same as that of S1 Fig. (TIF) [file pone.0133183.s002.tif]

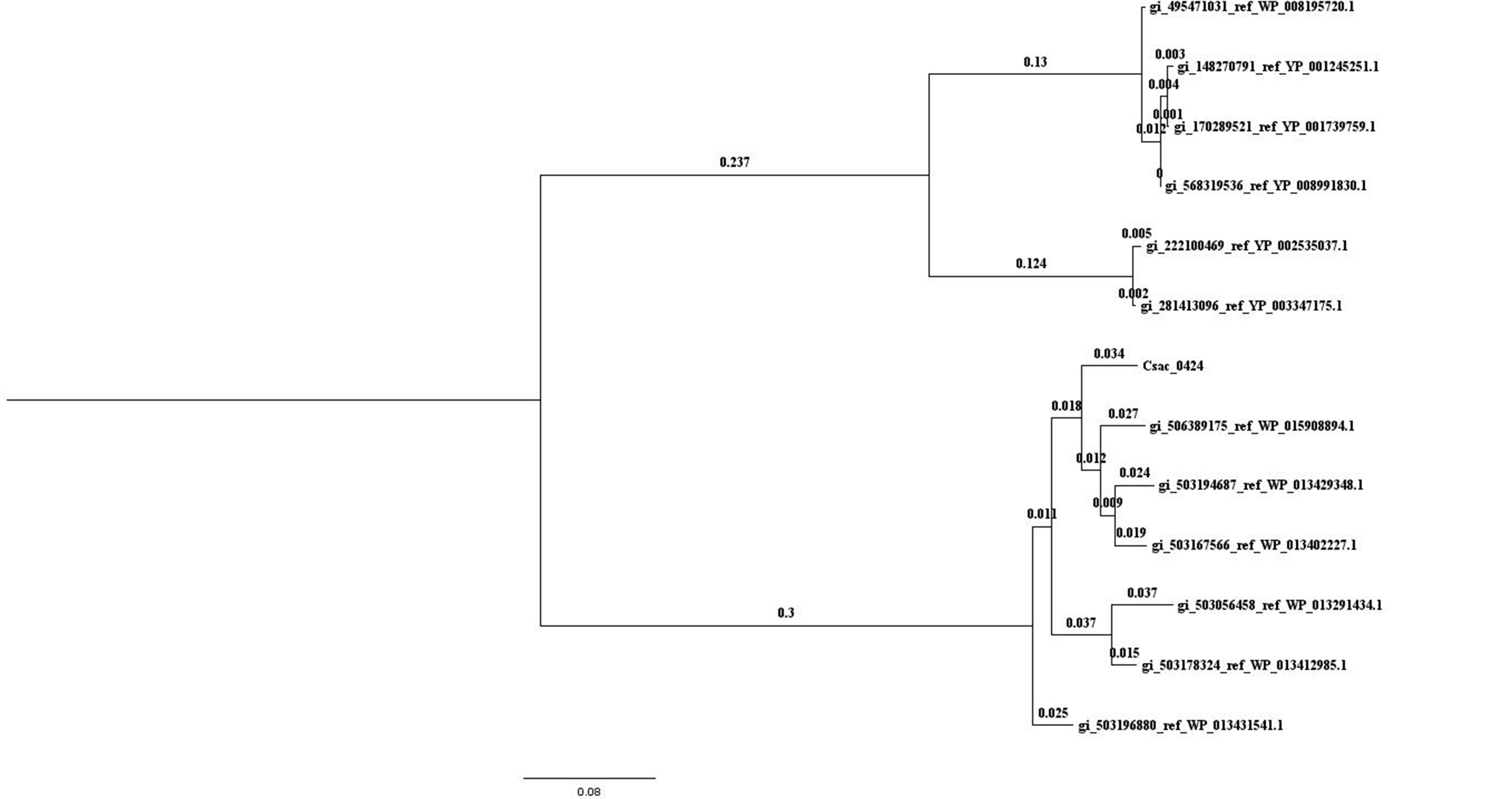

Supplement: S3 Fig — The corresponding NCBI RefSeq accession numbers and organisms taken for phylogenetic studies are as follows: C. saccharolyticus (Csac_0424); C. hydrothermalis, WP_013402227.1; C. bescii, WP_015908894.1; C. kronotskyensis, WP_013429348.1; C. kristjanssonii, WP_013431541.1; C. owensensis, WP_013412985.1; C. obsidiansis, WP_013291434.1; Thermotoga sp. EMP, WP_008195720.1; Thermotoga sp. RQ2, YP_001739759.1; Thermotoga petrophila RKU-1, YP_001245251.1; Thermotoga naphthophila RKU-10, YP_003347175.1; Thermotoga maritima MSB8, YP_008991830.1; Thermotoga neapolitana DSM 4359, YP_002535037.1. (TIF) [file pone.0133183.s003.tif]

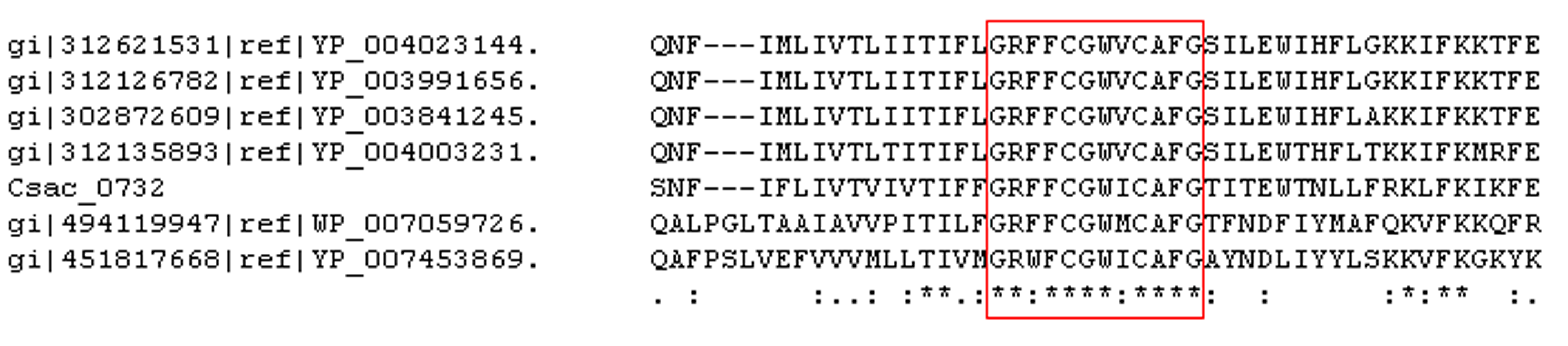

Supplement: S4 Fig — The corresponding NCBI RefSeq accession numbers and organisms are C. saccharolyticus (Csac_0732); C. obsidiansis OB47, YP_003841245.1; C. kronotskyensis 2002, YP_004023144.1; C. hydrothermalis 108, YP_003991656.1; C. owensensis OL, YP_004003231.1; Clostridium carboxidivorans, WP_007059726.1; Clostridium saccharoperbutylacetonicum N1-4(HMT), YP_007453869.1. (TIF) [file pone.0133183.s004.tif]

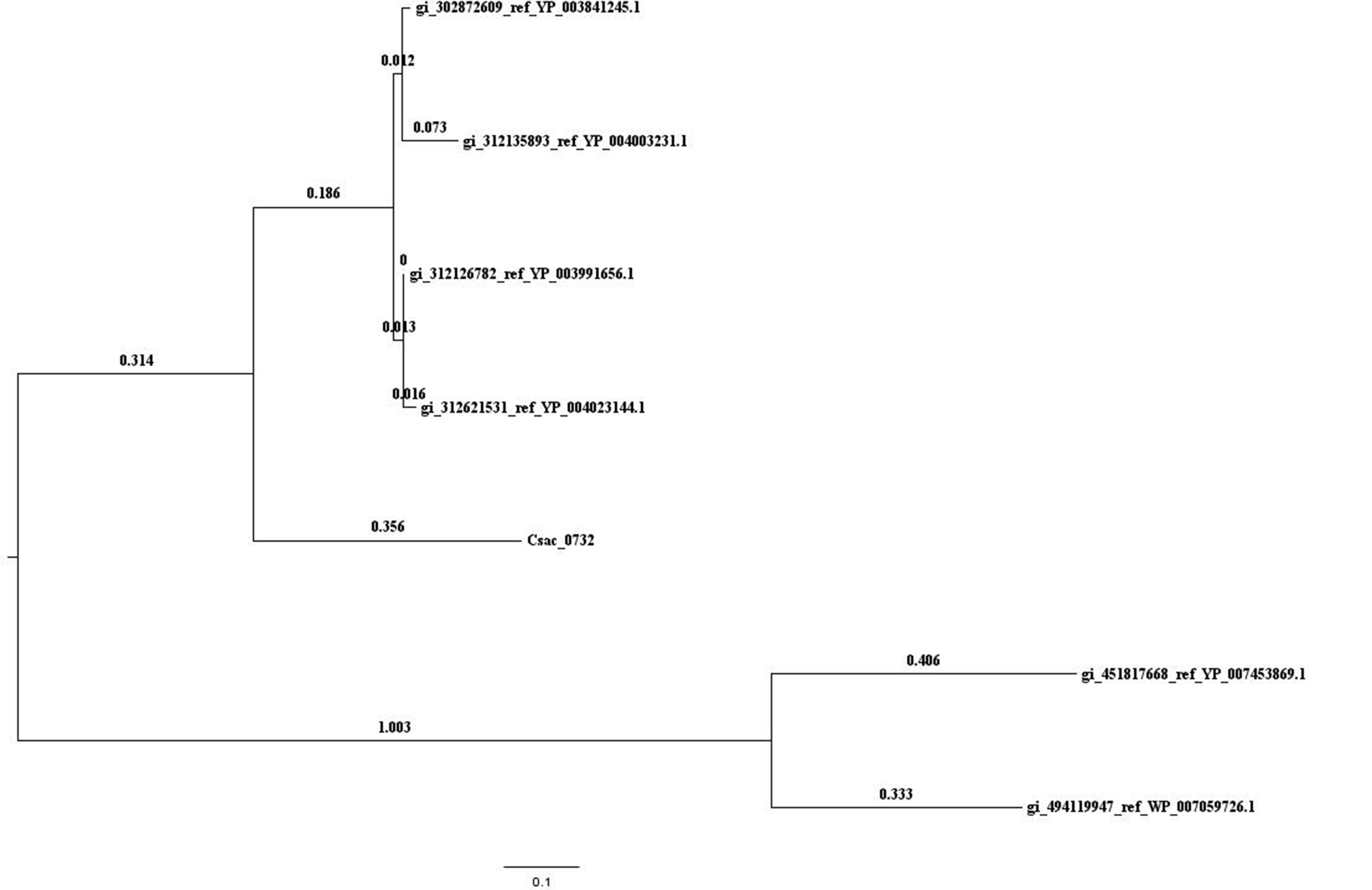

Supplement: S5 Fig — The corresponding NCBI RefSeq accession numbers and organisms taken for phylogenetic study of this hypothetical protein is same as that of S4 Fig. (TIF) [file pone.0133183.s005.tif]

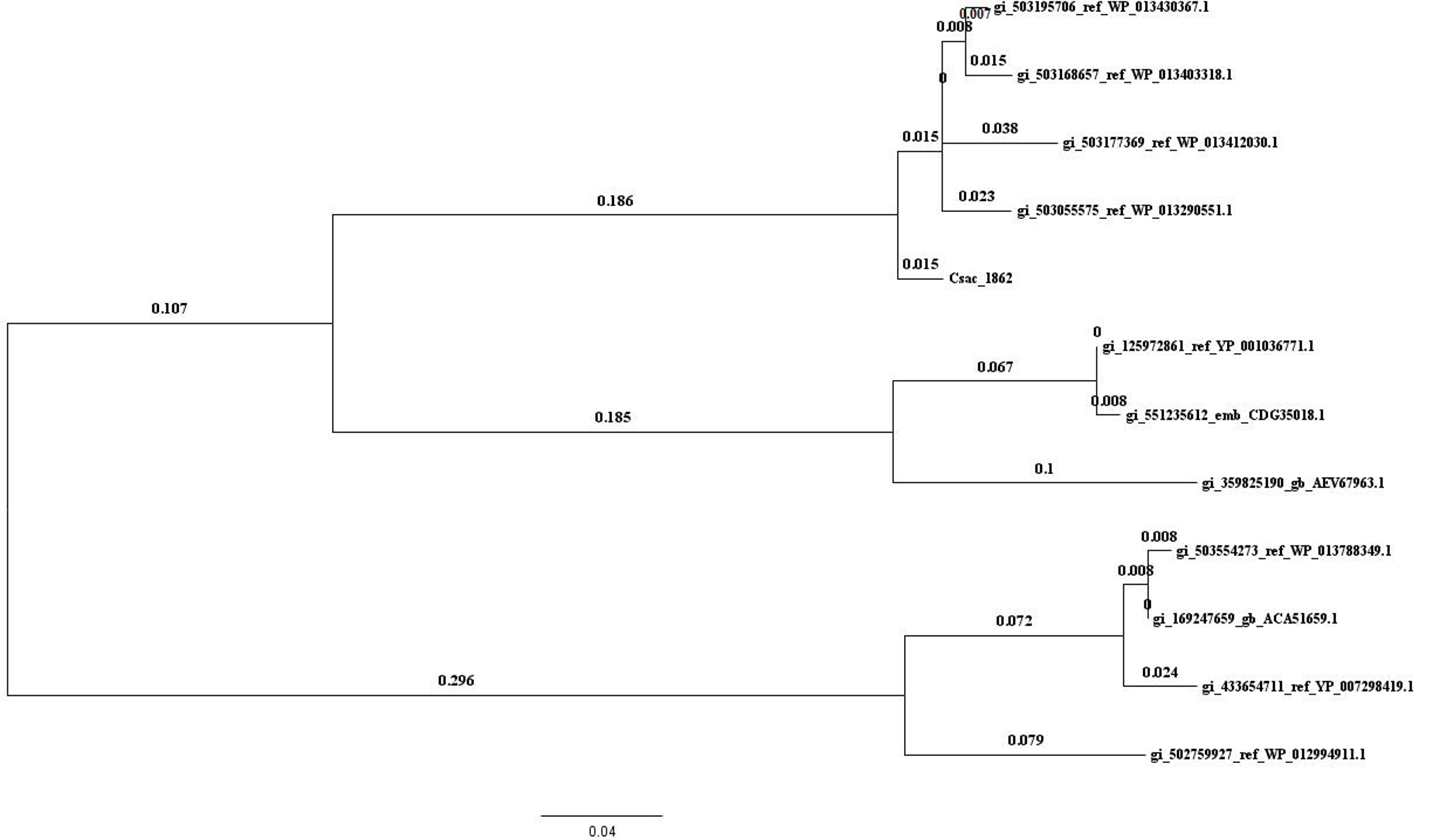

Supplement: S6 Fig — The corresponding NCBI RefSeq accession numbers and organisms taken for phylogenetic study are as follows: C. saccharolyticus (Csac_1862); Caldicellulosiruptor (multispecies), WP_013430367.1 and WP_013403318.1; C. obsidiansis, WP_013290551.1; C. owensensis, WP_013412030.1; Clostridium clariflavum DSM 19732, AEV67963.1; Clostridium thermocellum BC1, CDG35018.1; Clostridium thermocellum ATCC 27405, YP_001036771.1; Thermoanaerobacterium saccharolyticum JW/SL-YS485, ACA51659.1; Thermoanaerobacterium xylanolyticum, WP_013788349.1; Thermoanaerobacterium thermosaccharolyticum M0795, YP_007298419.1; Thermoanaerobacter, WP_012994911.1. (TIF) [file pone.0133183.s006.tif]

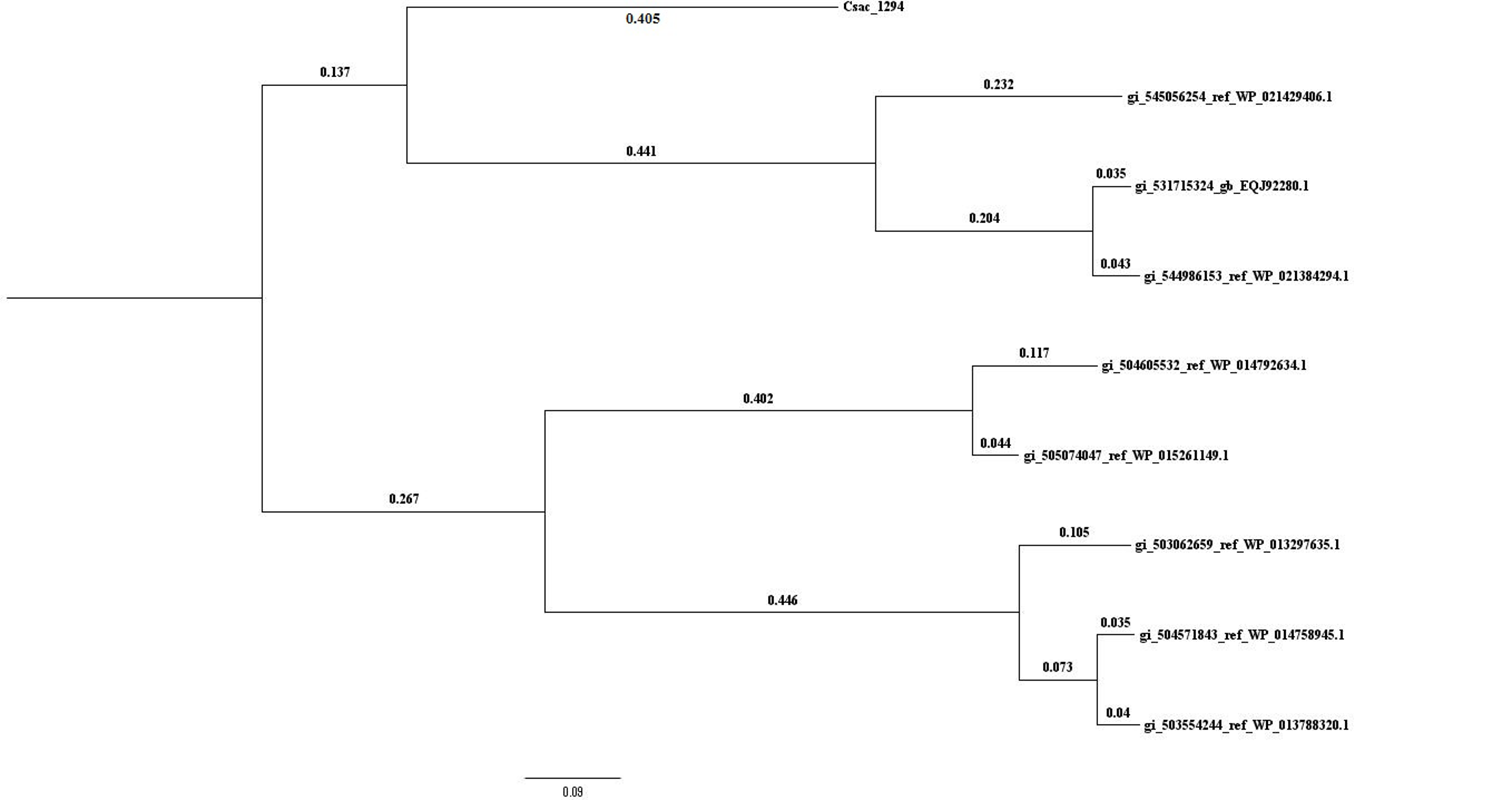

Supplement: S7 Fig — The corresponding NCBI RefSeq accession numbers and organisms are: C. saccharolyticus (Csac_1294); Peptoclostridium difficile, WP_021384294.1; Clostridium difficile P50, EQJ92280.1; Clostridium bifermentans, WP_021429406.1; Thermoanaerobacterium xylanolyticum, WP_013788320.1; Desulfitobacterium dichloroeliminans, WP_015261149.1; Thermoanaerobacterium saccharolyticum, WP_014758945.1; Thermoanaerobacterium thermosaccharolyticum, WP_013297635.1; Desulfitobacterium dehalogenans, WP_014792634.1. (TIF) [file pone.0133183.s007.tif]

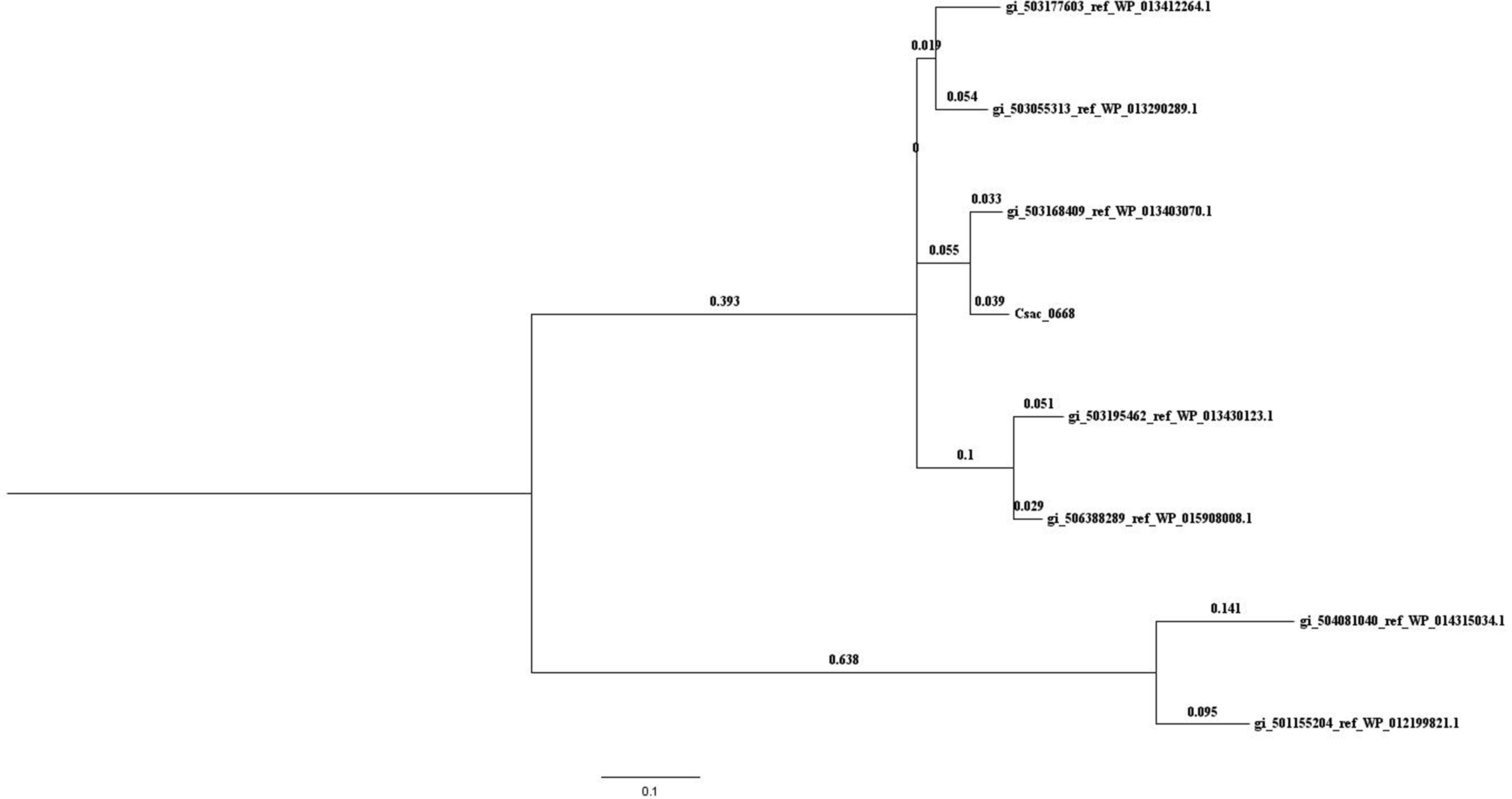

Supplement: S8 Fig — The corresponding NCBI RefSeq accession numbers and organisms are: C. saccharolyticus (Csac_0668); C. hydrothermalis, WP_013403070.1; C. bescii, WP_015908008.1; C. owensensis, WP_013412264.1; C. obsidiansis, WP_013290289.1; C. kronotskyensis, WP_013430123.1; Lachnoclostridium phytofermentans, WP_012199821.1; Clostridium sp. BNL1100, WP_014315034.1. (TIF) [file pone.0133183.s008.tif]
