# Supplementary material for: Genome Wide Re-Annotation of Caldicellulosiruptor saccharolyticus with New Insights into Genes Involved in Biomass Degradation and Hydrogen Production
Source: PLoS One. 2015 Jul 21;10(7):e0133183. doi: 10.1371/journal.pone.0133183 (PMC4510573; doi:10.1371/journal.pone.0133183)
Supplement: S7 Table — (DOCX) [file pone.0133183.s015.docx]

| **S. No.** | **Software name** | **Accuracy of prediction** | **Sensitivity** | **Specificity** | **Empiric ROC Area** |
| --- | --- | --- | --- | --- | --- |
|  | PSI- BLAST | 99% | 100% | 0% | 0.995 |
|  | BLASTO | 78.9% | 100% | 18.5% | 0.633 |
|  | Pfam | 91.5% | 100% | 13.7% | 0.885 |
|  | ANNIE | 94.5% | 100% | 20.3% | 0.744 |
|  | InterProScan | 93.7% | 100% | 25.2% | 0.893 |
|  | Average | 91.52% | 100% | 15.6% | 0.83 |

**Table S7:** List of accuracy, sensitivity, specificity and Empiric ROC area of five bioinformatics tools used for predicting functions of *Caldicellulosiruptor saccharolyticus* obtained after ROC analysis
