# Supplementary material for: Genome Wide Re-Annotation of Caldicellulosiruptor saccharolyticus with New Insights into Genes Involved in Biomass Degradation and Hydrogen Production
Source: PLoS One. 2015 Jul 21;10(7):e0133183. doi: 10.1371/journal.pone.0133183 (PMC4510573; doi:10.1371/journal.pone.0133183)
Supplement: S8 Table — (DOCX) [file pone.0133183.s016.docx]

**Table S8**: Synteny conservation of predicted Fe-S clusters and **CRISPR-associated protein genes across the members of genus *Caldicellulosiruptor.***

**Each row shows genes involved in the synteny group between *Caldicellulosiruptor saccharolyticus* and other species from the genus *Caldicellulosiruptor* and the columns depicts the names of the syntenic genes from each species.**

| ***C. saccharolyticus*** | ***C. bescii*** | ***C. hydrothermalis*** | ***C. kristjanssonii*** | ***C. kronotskyensis*** | ***C. lactoaceticus 6A (WGS)*** | ***C. obsidiansis*** | ***C. owensensis*** |
| --- | --- | --- | --- | --- | --- | --- | --- |
| Csac_0732  (4Fe-4S binding domain) |  | Calhy_0546  ( FMN-binding domain-containing protein) |  | Calkro_0425  ( FMN-binding domain-containing protein) |  | COB47_1994  ( FMN-binding domain-containing protein) | Calow_1902  ( FMN-binding domain-containing protein) |
| Csac_0668  (BFD domain-containing protein (2Fe-2S)-binding domain-containing protein) | Athe_1566  (BFD domain-containing protein (2Fe-2S)-binding domain-containing protein) | Calhy_1170  (BFD domain-containing protein (2Fe-2S)-binding domain-containing protein) |  | Calkro_1148  ((2Fe-2S)-binding domain-containing protein) |  | COB47_0988  (BFD domain-containing protein (2Fe-2S)-binding domain-containing protein) | Calow_1344  (BFD domain-containing protein (2Fe-2S)-binding domain-containing protein) |
| Csac_0050  (CRISPR-associated protein) |  |  | Calkr_2592  ( CRISPR-associated protein, APE2256 family) |  | CallaDRAFT_0409,  CallaDRAFT_0767  ( CRISPR-associated protein, APE2256 family) |  |  |
| Csac_0059  (CRISPR-associated protein) | Athe_2661  (CRISPR-associated helicase Cas3) |  | Calkr_2533  (CRISPR-associated HD domain-containing protein) |  | CallaDRAFT_0772  (CRISPR-associated helicase Cas3) |  |  |
| Csac_0312  (CRISPR-associated protein Cas6) | Athe_0121  (CRISPR-associated protein Cas6) | Calhy_2495  (CRISPR-associated protein Cas6) | Calkr_2425  (CRISPR-associated protein Cas6) | Calkro_2446  (CRISPR-associated protein Cas6) |  | COB47_0114  (CRISPR-associated protein Cas6) | Calow_0066  (CRISPR-associated protein Cas6) |
| CALS8_0358  (CRISPR-associated protein Cas6) | Athe_0121  (CRISPR-associated protein Cas6) | Calhy_2495  (CRISPR-associated protein Cas6) | Calkr_2425  (CRISPR-associated protein Cas6) | Calkro_2446  (CRISPR-associated protein Cas6) |  | COB47_0114  (CRISPR-associated protein Cas6) | Calow_0066  (CRISPR-associated protein Cas6) |
| CALS8_0367  (CRISPR-associated protein cas2) | Athe_0128,  Athe_2658  (CRISPR-associated protein cas2) | Calhy_2488  Calhy_2541  (CRISPR-associated protein cas2) | Calkr_2416  Calkr_2530  Calkr_2578  (CRISPR-associated protein cas2) | Calkro_2485  (CRISPR-associated protein cas2) | CallaDRAFT_0775  (CRISPR-associated protein cas2) | COB47_0121  COB47_2273  (CRISPR-associated protein cas2) | Calow_0074  (CRISPR-associated protein cas2) |
| CALS8_2622  (CRISPR-associated protein cas2) | Athe_0128  Athe_2658  (CRISPR-associated protein cas2) | Calhy_2488  Calhy_2541  (CRISPR-associated protein cas2) | Calkr_2416  Calkr_2530  Calkr_2578  (CRISPR-associated protein cas2) | Calkro_2485  (CRISPR-associated protein cas2) | CallaDRAFT_0775  (CRISPR-associated protein cas2) | COB47_0121  COB47_2273  (CRISPR-associated protein cas2) | Calow_0074  (CRISPR-associated protein cas2) |
